# Can circulating microRNAs predict colorectal cancer? Results from a nested case–control study of pre-diagnostic serum samples from two prospective biobanks

**DOI:** 10.1186/s12885-025-13854-1

**Published:** 2025-03-13

**Authors:** Eva Hofsli, Pål Sætrom, Eivind Ness-Jensen, Helja-Marja Surcel, Robin Mjelle

**Affiliations:** 1 Department of Clinical and Molecular Medicine, NTNU, Norwegian University of Science and Technology, Trondheim, Norway; 2 Department of Oncology, St. Olav’s Hospital, Trondheim University Hospital, Trondheim, Norway; 3 HUNT Research Centre, Department of Public Health and Nursing, NTNU, Norwegian University of Science and Technology, Levanger, Norway; 4 Department of Medicine, Levanger Hospital, Nord-Trøndelag Hospital Trust, Levanger, Norway; 5 Department of Molecular Medicine and Surgery, Karolinska Institutet and Karolinska University Hospital, Stockholm, Sweden; 6 St. Olavs Hospital HF, Sentral Stab, Trondheim, NO-7006 Norway; 7 Department of Computer Science, NTNU, Norwegian University of Science and Technology, Trondheim, Norway; 8 K.G.Jebsen Center for Genetic Epidemiology, NTNU – Norwegian University of Science and Technology, Trondheim, NO-7491 Norway; 9 Biobank Borealis of Northern Finland, Oulu University Hospital, Oulu, Finland; 10 Faculty of Medicine, University of Oulu, Oulu, Finland; 11 Department of Pathology, St.Olav’s Hospital, Laboratoriesenteret 4, Etg Erling Skjalgssons Gate 1, Trondheim, 7030 Norway; 12 HUNT Center for Molecular and Clinical Epidemiology, Department of Public Health and Nursing, NTNU, Norwegian University of Science and Technology, Trondheim, Norway

**Keywords:** Biomarkers, MicroRNAs, Colorectal Neoplasms, Diagnosis, Serum

## Abstract

**Background:**

This study aimed to investigate the potential of circulating small RNAs (sRNAs) as predictive biomarkers for future colorectal cancer (CRC). The study analyzed serum samples from pre-diagnostic CRC patients in two prospective biobanks.

**Methods:**

Serum samples from 142 pre-diagnostic CRC patients, from the Finnish Maternity Cohort (FMC) and The HUNT Study (HUNT2), were subjected to small RNA sequencing. The study compared sRNA expression in CRC cases with controls, considering diverse sRNA classes.

**Results:**

Analysis revealed diverse miRNA expression patterns with notable variations in future metastatic cases. Specifically, miR-223-3p and miR-21-5p showed significant up-regulation in future metastatic cases in the FMC cohort. Consistent changes were observed across cohorts, with miR-584-5p, miR-30c-5p, miR-146a-5p, miR-10a-5p, and miR-1306-5p showing up-regulation in future metastatic cases.

**Conclusions:**

The study identified potential serum miRNA biomarkers associated with metastatic CRC, though statistical significance varied. These findings contribute to the understanding of miRNA profiles in pre-diagnostic CRC patients, emphasizing the need for further exploration of non-invasive biomarkers in large prospective studies.

**Supplementary Information:**

The online version contains supplementary material available at 10.1186/s12885-025-13854-1.

## Background

Colorectal cancer (CRC) is the third most common cancer worldwide, with 1.9 million new cases and almost 1 million deaths from CRC each year [1]. CRC is particularly suitable for screening because most cases arise from adenomas, non-malignant precursor lesions [2–4]. Early detection is the key to improved survival since most early-stage CRC cases survive [5]. No circulating CRC screening biomarkers have been translated to the clinic or reached advanced clinical trials. Today, screening of risk groups by using immunochemical fecal occult blood tests (iFOBTs) followed by colonoscopy is the gold standard for CRC detection. However, iFOBTs are associated with false positive diagnoses [2, 6], and the impact of iFOBTs in reducing mortality remains unclear [7–10]. Further, colonoscopy is generally regarded as an expensive and demanding procedure. Non-invasive biomarkers, such as molecular markers in blood, may instead be alternatives or supplements to fecal blood tests and colonoscopy-based screening. Among these, circulating microRNAs (miRNAs) are particularly promising due to their stability in biofluids, ease of detection, and potential tumor specificity [11, 12]. Unlike many other biomolecules, miRNAs are resistant to degradation by enzymes, making them ideal candidates for blood-based biomarkers [13]. They can be measured using highly sensitive and specific methods, such as quantitative PCR and next-generation sequencing [14]. Moreover, miRNA expression profiles reflect tumor biology, as certain miRNAs are actively released by cancer cells and play roles in CRC progression and metastasis [15, 16]. Our group has previously shown that circulating miRNAs can differentiate CRC cases from healthy controls, particularly in patients with metastatic disease [17, 18], further indicating that miRNAs are indeed suited as biomarkers in CRC. However, no consensus miRNA-signature has been established that can predict CRC across cohorts, although several individual studies have provided promising results [17–20].

In this study, we aimed to investigate if miRNA levels in serum could predict future CRC diagnosis. 142 pre-diagnostic serum samples from two prospective biobanks were analyzed and the expression of 329 miRNAs was compared between future cases and healthy controls.

## Methods

### Study samples

The HUNT Study (HUNT) [21], is a comprehensive health survey conducted among the residents aged 20 years or older in the northern region of Trøndelag. Over the course of four surveys (HUNT 1–4), more than 120,000 individuals have actively participated by responding to questionnaires and providing blood samples. Within HUNT2, a nested case–control study was designed, comprising 96 incident cases of individuals who developed CRC after donating blood specimens between 1995 and 1997. Additionally, 96 controls were selected, matched based on sex and age. The identification of incident CRC cases, as well as the TNM staging, was accomplished by linking the participants' data with the Cancer Registry of Norway. The blood specimens collected during the study were stored at a temperature of -80 °C.

The Finnish Maternity Cohort (FMC) [22] comprises 2 million 1st trimester serum samples collected from 1 million women for 33 years, since 1983. Since FMC comprises samples from pregnant women, this biobank represents a unique opportunity to investigate CRC in young patients as well as in women. The median age of this population is about 30 years. A nested case–control study was designed, comprising 45 incident cases of CRC in women who developed CRC after donating blood specimens between 1986 and 2014. The identification of incident CRC cases, as well as the TNM staging, was accomplished by linking the participants' data with the Cancer Registry of Finland.

### RNA isolation, library preparation and sequencing

RNA was isolated from 200uL serum using the miRNeasy Serum/Plasma Kit (ID: 217,184). Small RNA sequencing libraries were prepared using NEXTFLEX® Small RNA-Seq Kit v3 for Illumina® Platforms, following the protocol. 10.5 ul of RNA was used as input. The libraries were amplified for 18 PCR cycles. The libraries were sequenced on a HiSeq4000 machine from Illumina using 50 nts single end reads.

### Calibrator RNAs

Synthetic calibrator RNAs were added during the first ligation step of the library preparation protocol as previously described [18].

### Data processing

The sequence data were processed as previously outlined by Mjelle et al. [23]. In short, trimmed sequence were collapsed using FASTX COLLAPSER tool (http://hannonlab.cshl.edu/fastx_toolkit/) and mapped to the human (hg38) genome using BOWTIE2 (22388286), allowing for up to 10 alignments per read. Reads overlapping with mature miRNA loci were identified using htseq-count from the HTSEQ PYTHON package (35561197). Mature miRNAs and non-coding RNAs were annotated using miRBase (Release 21, 2014) [24] and RNA Central (http://rnacentral.org) [25], respectively.

### Sequencing data analysis

Differentially expressed small RNAs were determined by using limma-voom [26] in R. The *p*-values from the limma-model were adjusted for multiple testing using the Benjamini–Hochberg procedure. Small RNAs with at least 1 count in 25% of the data were considered in the analysis. The *calcNormFactors* of the calibrator RNAs were used as normalization factors in the DGE-object of the small RNAs. For the analyses of the FMC samples, the limma-models were adjusted for sample-age due to the large interval in sample collection dates (1983–2014). In both cohorts, the limma-model incorporated age as a covariate. Additionally, for the HUNT cohort, the model was adjusted for sex.

## Results

### Sequencing statistics

In total, 142 future CRC patients from two prospective cohorts were analyzed. The Finnish Maternity Cohort (FMC) consisted of 46 cases and 49 controls, and the second, The HUNT Study (The HUNT2 Survey), consisted of 96 cases and 96 controls. On average across all cases and controls, 4.8 million reads aligned to the human genome, of which 1.1 million reads overlapped human miRNAs (Supplementary Fig. 1A-B). We detected small RNAs (sRNAs) from different RNA classes of which miRNAs, long non-coding RNAs (lncRNAs), ribosomal RNAs (rRNAs), transfer RNAs (tRNAs) and Y-RNAs were the most abundant RNA classes (Supplementary Fig. 2A-B). The processed reads were enriched for 22-nucleotides (nt) fragments, corresponding to mature miRNAs, in addition to 30-nt-long tRNA fragments (Supplementary Fig. 1C-F). The number of aligned reads was even across samples for all cohorts although we did observe variations in the number of miRNA-reads and other RNA-classes across samples, likely due to differences in RNA-input levels (Supplementary Fig. 1A-B).

### Overview of cohorts and data

**Fig. 1:**
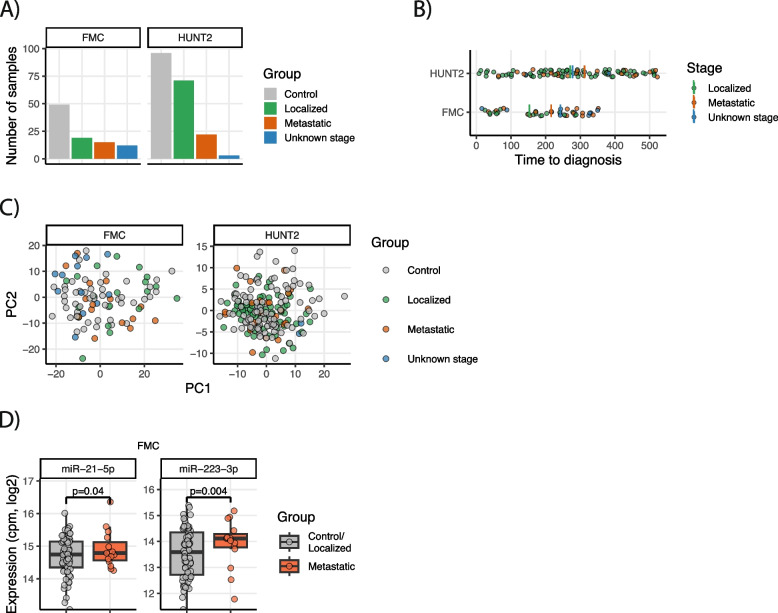
**A** Number of analyzed case and control samples stratified by TNM stage. Shown are the number of control samples, samples with localized disease (TNM I-II); metastatic disease (TNM III-IV); and samples with unknown stage. **B** Time from sample collection to cancer diagnosis (time-to-diagnosis) for all analyzed case samples, colored by TNM-stage. The mean time-to-diagnosis is indicated with a coloured vertical bar for each of the TNM-groups. **C** Principal component analysis of the miRNA expression data for the FMC and HUNT2 cohort, colored by sample group. **D** Significantly differentially expressed miRNAs in the FMC cohort. The boxplots compare the metastatic case samples to all controls and cases with localized disease. The *p*-values are the Benjamini-Hochberg-adjusted *p*-values from the limma-model in R

Of the 142 analyzed cases, 90 were from patients with localized disease (TNM stage I-II), 37 from patients with distant metastases (TNM IV) or regional lymph node metastases (TNM stage III) and 15 were from patients with unknown stage (Fig. 1A). The time between sample collection and the cancer diagnosis (time-to-diagnosis) varied from 8 to 522 days, with a mean time-to-diagnosis of 198 days for FMC and 281 days for HUNT2 (Fig. 1B).

### Differentially expressed miRNAs in pre-diagnostic serum samples

In total, 329 unique miRNAs were analyzed across the two cohorts. A principal component analysis of the miRNAs showed no clear separation between cases and controls or between the different stage groups, indicating lack of major miRNA changes between the groups (Fig. 1C). However, linear models revealed changes of individual miRNAs with respect to disease stage. First, each cohort was analyzed individually with respect to the following five contrasts: i) metastatic cases vs all controls; ii) localized cases vs all controls; iii) metastatic cases vs all controls and localized cases combined; iv) metastatic cases vs localized cases; v) all cases vs all controls. In the FMC cohort, two miRNAs, miR-223-3p and miR-21-5p, showed changes related to metastatic disease. Both miRNAs were significantly up-regulated in metastatic cases compared to the combined group of localized cases and all controls (Fig. 1D). Both miRNAs were also up-regulated between metastatic cases and controls only, however, the up-regulation was not significant after *p*-value adjustment (*p* = 0.12 and 0.21 for miR-223-3p and miR-21-5p, respectively). In the HUNT2 cohort, no significant miRNAs were detected for the abovementioned comparisons after *p*-value adjustment.

**Fig. 2:**
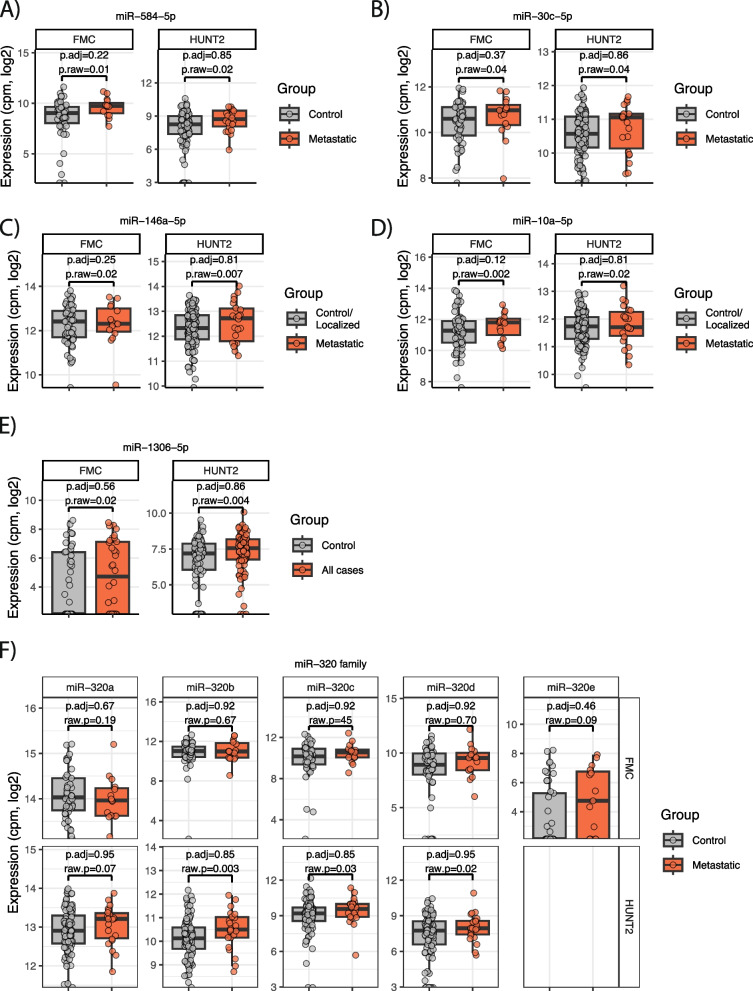
**A**-**B** MicroRNAs consistently up-regulated in metastatic cases vs all controls in the FMC and HUNT2 cohorts. Shown are the raw un-adjusted *p*-values (“p.raw”) and the Benjamini-Hochberg-adjusted *p*-values (“p.adj”) from the limma-model in R. **C-D** MicroRNAs consistently up-regulated in metastatic cases vs localized cases and all controls combined, in the FMC and HUNT2 cohorts. *P*-values as described in **A**)** E**) Expression of miR-1306-5p in all cases and all controls in FMC and HUNT2. *P*-values as described in **A**) **F**) Boxplot showing the expression of miRNAs within the miR-320 family in metastatic cases and all controls in the FMC- and HUNT2-cohorts. *P*-values as described in **A**)

Having shown that there were only small changes in miRNA expression within the individual cohorts, we instead compared fold-change values between FMC and HUNT2 to look for consistent changes between cohorts, focusing only on miRNAs with *p*-values less than 0.05 *before* *p*-value adjustment. We found that miR-584-5p and miR-30c-5p were up-regulated in metastatic cases vs all controls in both FMC and HUNT2 (Fig. 2A-B). Further, when comparing metastatic cases vs the group of controls and localized cases, miR-146a-5p and miR-10a-5p were detected as significantly up-regulated in metastatic cases in both cohorts before *p*-value adjustment (Fig. 2C-D). When comparing all cases vs all controls, one miRNA, miR-1306-5p, showed consistent up-regulation in both cohorts (Fig. 2E).

Our group has previously identified the miRNA family miR-320 as elevated in serum of metastatic CRC patients [17, 18]. We therefore wanted to specifically investigate miRNAs within this family. Interestingly, in the HUNT2 cohort, miR-320b is the most significant miRNA when comparing metastatic cases and all controls, and miR-320c and miR-320d were among the top 10 up-regulated miRNAs, all with significant *p*-values before *p*-value adjustment (Fig. 2F). In the FMC cohort, miR-320e was the most prominent miR-320 member, although not significant after *p*-value adjustment (Fig. 2F).

In addition to miRNAs, other sRNAs were tested in the aforementioned comparisons; however, no significant results were obtained.

To summarize, when comparing serum miRNA expression between prediagnostic CRC cases and healthy controls, we observed changes that were dependent on the stage of the disease and the time to diagnosis. Specifically, patients with advanced disease and those who were closer to diagnosis exhibited the most significant alterations in serum miRNAs when compared to healthy controls.

## Discussion

Previous studies analyzing samples collected *at diagnosis* suggest that serum miRNA-levels differ between CRC patients and healthy controls. However, no consensus miRNAs have been identified, which suggests that the presence of technical and biological differences between studies and cohorts impedes the identification of clinically relevant biomarkers. Considering the lack of consensus biomarkers at diagnosis, it is not surprising that we also fail to identify pre-diagnostic miRNAs with consistent changes across cohorts. Pre-diagnostic cancer samples are difficult to obtain, and large prospective biobanks with long follow-up are needed. Comprehensive clinical data are also required to identify the cancer-specific samples with information on stage and other relevant metadata. The FMC and HUNT cohorts are some of the few such biobanks and were chosen due to their availability to the research group and good clinical data.

The FMC and HUNT studies involve two distinct study populations. The FMC study consists of young females who had serum collected during pregnancy, while HUNT2 includes participants of various ages and both sexes. Since FMC consists of a more homogenous study population, it might be reasonable to assume that the biological variation in miRNA expression is smaller in FMC than in HUNT2. However, immune responses during pregnancy could preclude cancer-specific changes in serum miRNAs, masking biological signals that are present in HUNT2, leading to reduced consistency between the cohorts. The FMC samples were collected during a relatively long timespan and degradation of RNA could introduce technical biases that affect serum miRNA levels. However, there were no noticeable differences in RNA abundance and quality between samples, indicating that the quality and integrity of the measured RNA are robust across time (Supplementary Fig. 1B, D and F). This is particularly evident in the fragment length distribution, which remains relatively consistent across samples, indicating similar levels of degradation and a distinct miRNA-peak around 22 nts. Due to the low RNA-input levels, Nanodrop or Qubit measurements were not possible, but the fragment length analysis provides a good approximation of the RNA-quality.

Although only small changes in miRNA expression are observed for the two cohorts compared to controls, it is interesting to observe that the only significant changes were found in metastatic cases versus controls in the FMC cohort. A previous study on lung cancer showed that the most pronounced differences in pre-diagnostic miRNA-levels were for metastatic cases close to diagnosis [27]. In our study, slightly more consistency was found when grouping control samples and cases with localized disease together, indicating that the power to detect consistent and significant changes might be too low. Future studies that include more metastatic cases could potentially detect more significant changes. Also, having homogenous sample groups, with, for instance age and sex matched samples, would decrease variation and increase potential cancer-specific signals. Also, using samples from individuals that are at risk of having CRC, for instance with positive iFOBT tests, could further increase the chances of detecting cancer-related miRNAs. Given that we and other research groups have detected distinct miRNA-serum profiles at diagnosis, it is likely that these signals are indeed present also before diagnosis; the question remains how early they can be detected. However, identifying enough pre-diagnostic CRC cases is challenging, and requires large prospective biobanks, especially when subgrouping the participants into different risk groups.

Several of the identified miRNAs, including miR-223-3p, miR-21-5p, and miR-584-5p, have been implicated in CRC pathogenesis. miR-21-5p is one of the most well-established oncogenic miRNAs in CRC, promoting tumor growth, invasion [28, 29], and also previously shown to be up-regulated in serum of CRC patients [30]. MicroRNA miR-223-3p has been linked to inflammation-driven CRC progression, influencing pathways such as NF-κB and epithelial-mesenchymal transition [31, 32]. Although less studied in CRC, miR-584-5p has been suggested to play a role in tumor suppression by targeting oncogenic pathways in other cancers [33]. The dysregulation of these miRNAs in pre-diagnostic serum samples suggests their potential involvement in early tumor development or systemic immune responses, however, their role need to be further investigated in other cohorts.

New analysis-methods could identify biologically relevant signals in the data presented in the current study. Here, we rely on linear-models to compare differences in mean miRNA-expression between future CRC cases and controls. These methods require large differences and low variance between the groups for miRNAs to be regarded as significant. The data presented in the current study could be valuable to include in future meta-analyses as more pre-diagnostic sequencing experiments are being performed.

While our study did not identify a consistent pre-diagnostic miRNA signature across cohorts, the observed dysregulation of certain miRNAs in metastatic cases suggests that these biomarkers could complement current screening methods, particularly for identifying high-risk individuals. Future studies should explore whether combining miRNA profiling with established screening tools, such as fecal immunochemical tests (FIT) or colonoscopy, could enhance early detection rates. Additionally, machine learning approaches integrating multiple biomarkers, including miRNA panels, may help refine risk stratification strategies in prospective cohorts. Ultimately, large-scale validation studies in well-characterized pre-diagnostic samples are necessary before these biomarkers can be translated into clinical practice.

## Supplementary Information

Supplementary Material 1: Fig. 1.  A) Absolute number of sequencing reads for the HUNT2 data. “Total” is the total number of reads in the libraries; “SingleAligned” is reads that align at one position in the genome; “MultiAligned” is reads that align at multiple positions in the genome; “NotAligned” is reads that do not align to the human genome (NotAligned), Number of reads that overlap the different RNA classes are indicated by colour and RNA class name. B) Similar as in A) for the FMC-cohort.  C) Heatmap showing the number of reads (rpm-normalized) for different fragment lengths in the HUNT2-cohort. D)  Similar as in C) for the FMC-cohort. E) Relative number of reads for the main RNA-classes with respect to fragment length. F) Similar as in E) for the FMC-cohort.

Supplementary Material 2: Fig. 2. A) Relative number of reads overlapping the main RNA-classes in the HUNT2-cohorts. B) Similar as in A) for the FMC-cohort.

## Data Availability

Count matrices and metadata are available via github repository: https://github.com/MjelleLab/Prediagnostic-miRNA-profiling-in-CRC. Due to Norwegian law on sensitive data, raw data cannot be submitted to public repositories, however, raw data can be made available upon request to the corresponding author through research collaborations. Due to Norwegian law on sensitive data, raw data cannot be submitted to public repositories, however, raw data can be made available upon request to the corresponding author through research collaborations.
