# Supplementary figures and images for: Can circulating microRNAs predict colorectal cancer? Results from a nested case–control study of pre-diagnostic serum samples from two prospective biobanks
Source: BMC Cancer. 2025 Mar 13;25:455. doi: 10.1186/s12885-025-13854-1 (PMC11905635; doi:10.1186/s12885-025-13854-1)

A)

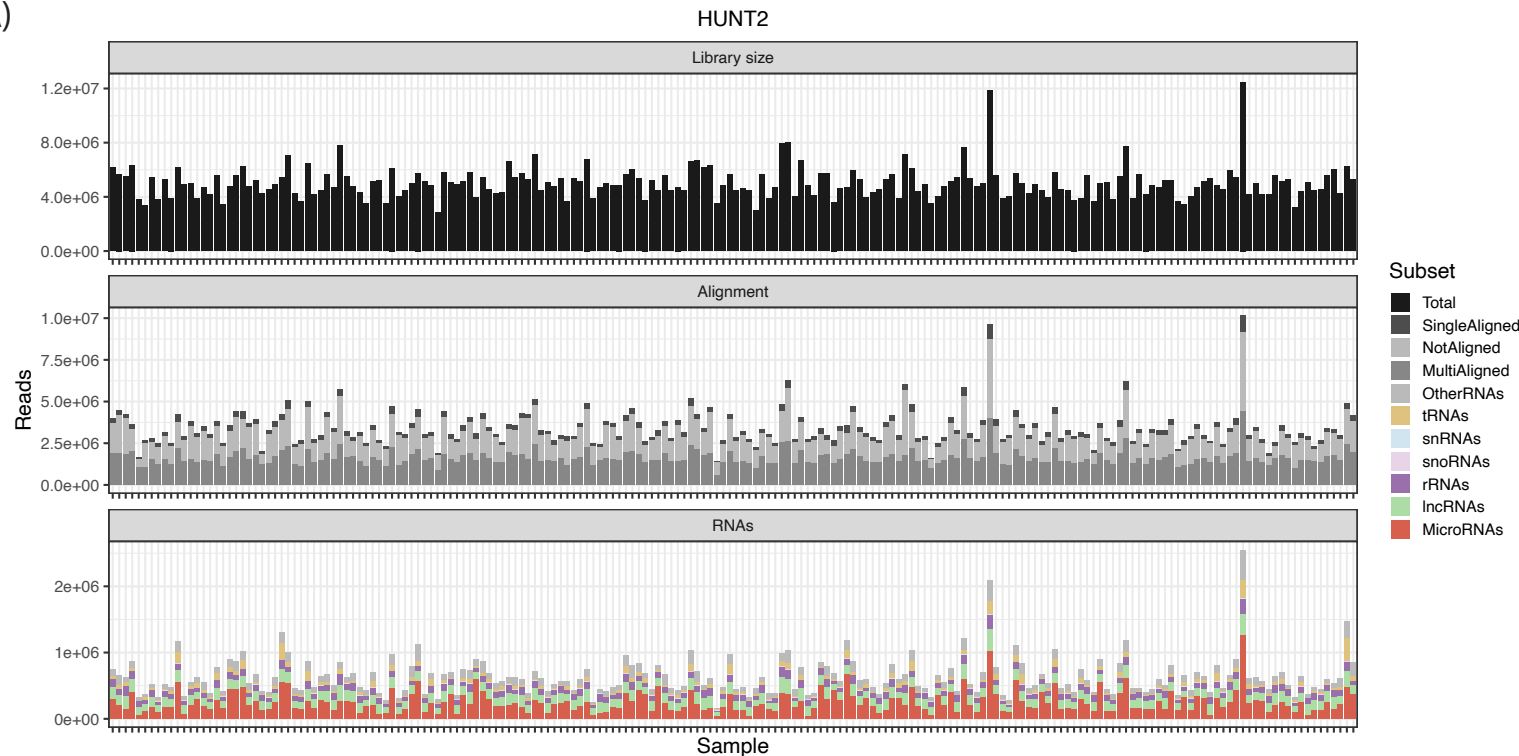

B)

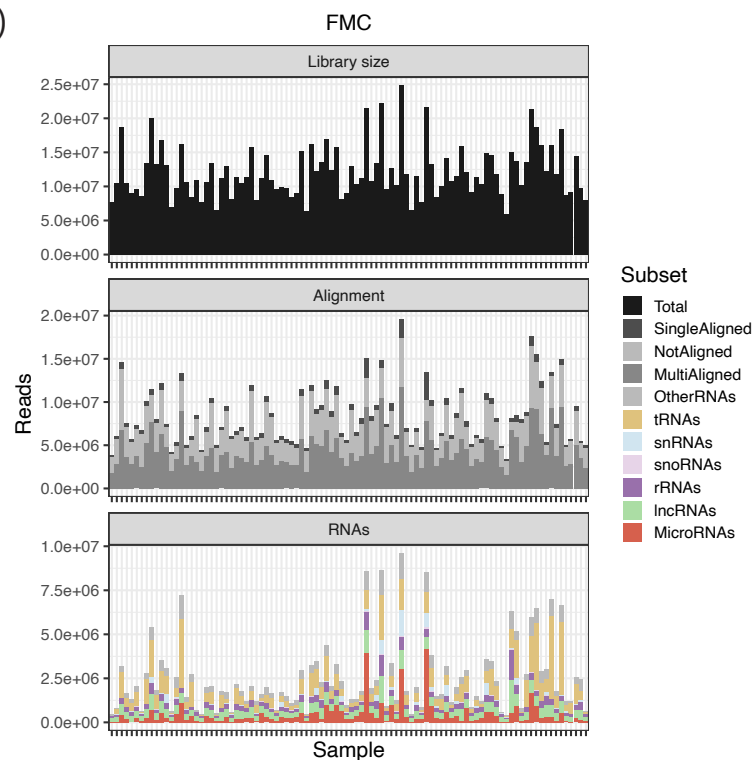

C)

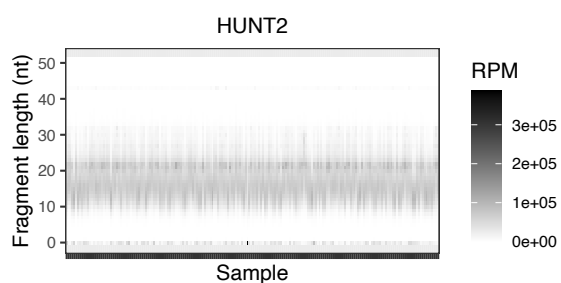

D)

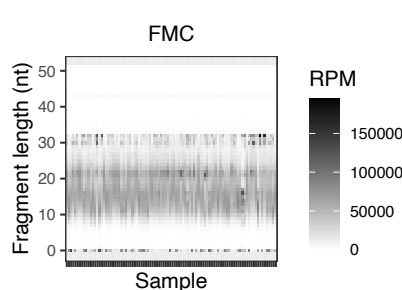

E)

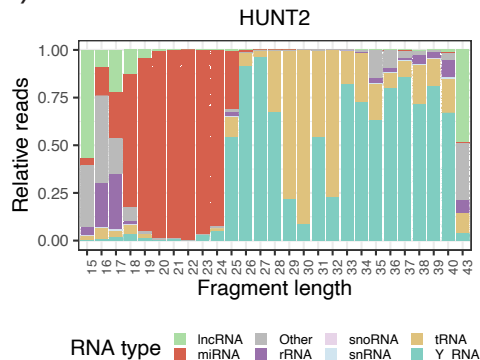

F)

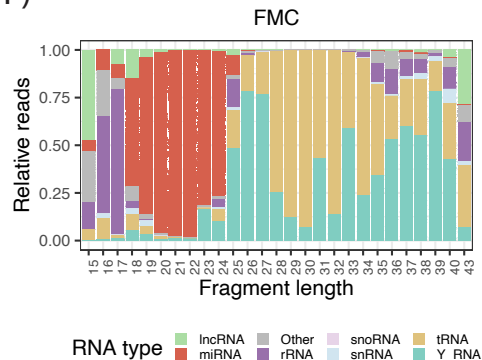

Supplement: Supplementary file 1 — Supplementary Material 1: Fig. 1. A) Absolute number of sequencing reads for the HUNT2 data. “Total” is the total number of reads in the libraries; “SingleAligned” is reads that align at one position in the genome; “MultiAligned” is reads that align at multiple positions in the genome; “NotAligned” is reads that do not align to the human genome (NotAligned), Number of reads that overlap the different RNA classes are indicated by colour and RNA class name. B) Similar as in A) for the FMC-cohort. C) Heatmap showing the number of reads (rpm-normalized) for different fragment lengths in the HUNT2-cohort. D) Similar as in C) for the FMC-cohort. E) Relative number of reads for the main RNA-classes with respect to fragment length. F) Similar as in E) for the FMC-cohort. [file 12885_2025_13854_MOESM1_ESM.pdf]

A)

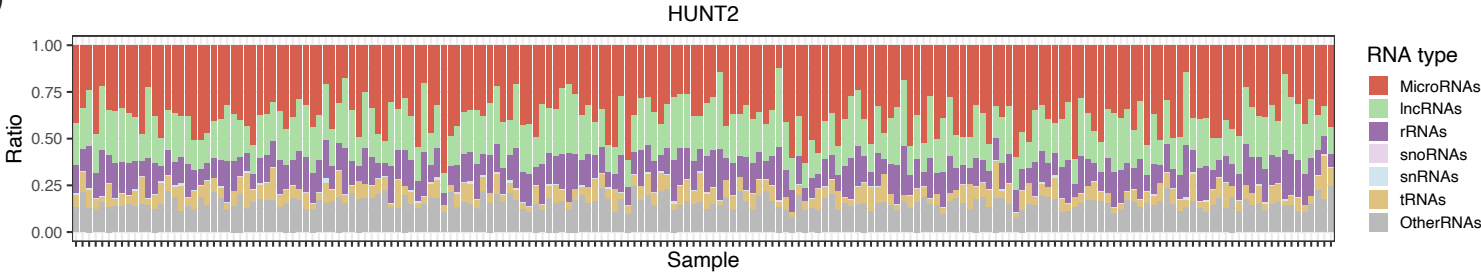

B)

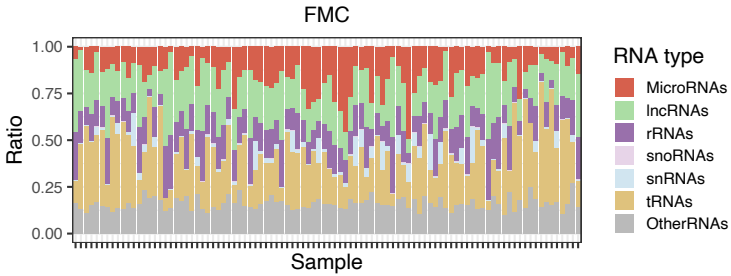

Supplement: Supplementary file 2 — Supplementary Material 2: Fig. 2. A) Relative number of reads overlapping the main RNA-classes in the HUNT2-cohorts. B) Similar as in A) for the FMC-cohort. [file 12885_2025_13854_MOESM2_ESM.pdf]
